# Classifying Human Voices by Using Hybrid SFX Time-Series Preprocessing and Ensemble Feature Selection

**DOI:** 10.1155/2013/720834

**Published:** 2013-10-29

**Authors:** Simon Fong, Kun Lan, Raymond Wong

**Affiliations:** ^1^Department of Computer and Information Science, University of Macau, Macau; ^2^School of Computer Science and Engineering, University of New South Wales, Kensington, NSW 2052, Australia

## Abstract

Voice biometrics is one kind of physiological characteristics whose voice is different for each individual person. Due to this uniqueness, voice classification has found useful applications in classifying speakers' gender, mother tongue or ethnicity (accent), emotion states, identity verification, verbal command control, and so forth. In this paper, we adopt a new preprocessing method named Statistical Feature Extraction (SFX) for extracting important features in training a classification model, based on piecewise transformation treating an audio waveform as a time-series. Using SFX we can faithfully remodel statistical characteristics of the time-series; together with spectral analysis, a substantial amount of features are extracted in combination. An ensemble is utilized in selecting only the influential features to be used in classification model induction. We focus on the comparison of effects of various popular data mining algorithms on multiple datasets. Our experiment consists of classification tests over four typical categories of human voice data, namely, Female and Male, Emotional Speech, Speaker Identification, and Language Recognition. The experiments yield encouraging results supporting the fact that heuristically choosing significant features from both time and frequency domains indeed produces better performance in voice classification than traditional signal processing techniques alone, like wavelets and LPC-to-CC.

## 1. Introduction

Unlike fingerprints, iris, retina, and facial feature, our voice is a kind of bodily characteristics that is useful in speaker identification but it remains relatively unexplored. Compared to other bodily features, voice is dynamic and complex, in the sense that a speech can be spoken in different languages, different tones, and in different emotions. Voice biometrics plays a central role in many biometrics applications such as speaker verification, authentication, and access control management. Furthermore voice classification potentially can apply to interactive-voice-response system for detecting the moods and tones of customers, thereby guessing if the calls are of complaints or complement, for example. More examples of voice classification have been described in our previous work [1] which attempted to classify voice data by using hierarchical time-series clustering methods. The clustering method only separates voice data into distinct groups without knowing the labels of the groups. Voice classification method trains and tests voice data into classes of known labels.

Voice classification has been studied intensively in the biometrics research community using digital signal processing methods. The signatures of the voice are expressed in numeric values in the frequency domain. There lie considerable challenges in attaining high accuracy in voice classification given the dynamic nature in the speech data, not only the contents within but also the diversity of human vocals and different ways of speeches. In this paper we tackle the classification challenges by modeling human voices as time-series in the form of stochastic signals. In contrast to deterministic signals that are rigidly periodic, stochastic signals are difficult to be modeled precisely by mathematical functions due to uncertainty in the parameters of the computational equations. Time-series of voice data are nonstationary, with their statistical characteristics change over time when spoken. As far as human voice is concerned, almost all of them are stochastic and nonstationary, meaning that their statistics are time dependent or time varying.

Given such temporal data properties, human voice that is acquired continually from the time domain would be in the form of random time-series that often has a single variable (amplitude in loudness) over time. It is believed that the statistical characteristics are changing over time during a speech but they may form some specific patterns, so some inherent information can be derived from the time-series that are useful for classification. Specifically we adopt a recent preprocessing methodology, called Statistical Feature Extraction (SFX) [2], that can effectively transform a univariate time-series voice data to a multivariate data while capturing the informative characteristics of the time-series. It is known that conventional data mining models can be deployed for classifying data with only multiple attributes. Previous work by other researchers who utilized wavelet transformation essentially converted temporal data to the representation of frequency domain format. For voice classification in this paper, elements of both time domain and frequency domain are used for obtaining the statistical characteristics of the time-series, and subsequently subject to model learning for classification that can be generically implemented by most of the available classification algorithms. 

Simulation experiments are carried out over four representative types of voice data or speeches being digitized for validating the efficacy of our proposed voice classification approach based on SFX and metaheuristic feature selection. This type of feature selection will find the optimal subset of features for inducing the classification model with the highest accuracy. The four types of testing data are deliberately chosen with the purpose of covering a wide range of possible voice classification applications, such as Female and Male (FM), Emotional Speech (ES), Speaker Identification (SI), and Language Recognition (LR). Given the multiattributes which are derived from the original time-series via the preprocessing step, feature selection (FS) techniques could be applied prior to training a classification model. Our results indicate that superior performance could be achieved by using SFX and FS together over the original time-series for voice classification. The improvements are consistent over the four testing datasets with respect to the major performance indicators.

The rest of the paper is structured as follows: The previous works on classifying voice data are reviewed in Section 2; specifically their time-series transformation and feature extraction techniques are highlighted. Our proposed voice classification model which converts time-series voice data to its encoded vector representation via statistical and spectral analysis is described in detail in Section 3. A set of comparative experiments is performed by using four kinds of voice datasets, and they are reported in Section 4. Results that reinforce the efficacy of our new approach are shown in Section 5. The performance evaluation is all-rounded by considering accuracy, Kappa statistic, precision, recall, *F*-measure, ROC area under curve, and time cost for each dataset.  Section 6 concludes this research work and suggests some future works.

## 2. Related Work

Human voice is stochastic, nonstationary, and bounded in frequency spectrum; hence some suitable features could be quantitatively extracted from the voice data for further processing and analysis. Over the years, different attempts have been made by previous researchers who used a variety of time-series preprocessing techniques as well as the core classification algorithms for extracting acoustic features from the raw time-series data. Their performances, however, vary.

### 2.1. Feature Extraction on Voice Data

Some useful features selected for the targeted acoustic surveillance are [3] weighted average delta energy (Δ_*E*_), LPC spectrum flatness (*F*_LPC_), FFT spectrum flatness (*F*_FFT_), zero crossing rate (*R*_ZC_), harmonicity (*H*), mid-level crossing rate (*R*_MC_), and peak and valley count rate (*R*_PV_). The classifier model used by the authors is the sliding window Hidden Markov Model (HMM). They obtained an average error rate at the range of 5%–20%. Peeters discovered more detailed acoustic features for sound description [4]. These features can be roughly grouped into temporal, energy, spectral, harmonic, perceptual, and various features. The limitation is the expensive time and space costs of computation for such full kind of feature extraction.

In the research community of signal processing, the most widely used methods for voice/speech feature extraction are Linear Prediction Coding or Linear Prediction Coefficient (LPC), Cepstral Coefficient or Cepstrum Coefficient (CC), and Mel Frequency Cepstral Coefficient (MFCC). LPC consists of finding a time-based series of *n*-pole infinite impulse response (IIR) filters whose coefficients better adapt to the formants of a speech signal. The main idea behind LPC is that a sample of speech can be approximated as a linear combination of past speech samples [5]. The methods for calculating LPCs include covariance method, autocorrelation (Durbin) method, lattice method, inverse filter formulation, spectral estimation formulation, maximum likelihood method, and inner product method [6].

As a general practice of pattern recognition, the final predictor coefficients are never applied because of the high variance. Instead, cepstral coefficients [7] are introduced for transforming the LPC predictor coefficients to those with more robust property. Cepstral coefficients are the inverse Fourier transform representation of the log magnitude of the spectrum. The cepstral series represents a progressive approximation of the envelope of the signal [8]. MFCC offers the best performance within six coefficients (the other five coefficients are Linear Prediction Coefficient, Linear Prediction Cepstral Coefficient, Linear Frequency Cepstral Coefficient, and Reflection Coefficient) [9]. MFCC divided the speech into frames (typically 20 ms for each frame), applied Discrete Fourier Transformation over every frame, retained the logarithm of the amplitude spectrum, smoothed the spectrum, and applied Discrete Cosine Transform [10]. Several modified MFCC methods are shown having better performance in some cases. One of them is weighted MFCC. To reduce the dimensions of feature vector while still retaining the advantages of delta and double delta features, the weighted MFCC coefficients equal the sum of MFCC coefficients, *p* times Delta features and, *q* times double Delta features, where *p* and *q* are weights in real numbers [11]. An enhanced technique for feature recognition using Improved Features for Dynamic Time Warping (DTW) was applied as a classifier; the accuracy was between 85% and 98%. Zhou et al. designed a new Kullback-Leibler distance (KLD) based weighting Perceptual Linear Prediction (PLP) algorithm for MFCC. The KLD is defined as the distance of two continuous functions; it is a measure between reality distribution *p* and approximating model *q*. The weight is the reciprocal of this distance [12]. The word error rate was below 25%. 

Similar to LPC and MFCC, PLP modifies the short-term spectrum of the speech by several psychophysically based transformations. The basic steps of PLP contain spectral analysis, critical-band spectral resolution, equal-loudness preemphasis, intensity-loudness power law, autoregressive modeling, and practical considerations [13]. But PLP is vulnerable when spectral values are modified by the frequency response of the communication channel. Thus, by employing relative spectra filtering of log domain coefficients (RASTA), we make PLP more robust to these distortions [14].

Tsrrneo Nitta used multiple mapping operators to extract topological structures, hidden in time spectrum patterns. Linear algebra is the main technique. Karhunen-Loeve transformation and linear discriminant analysis were the feature extraction methods [15]. The error rate was lower than 30%. Lee et al. proposed a new feature extraction method called independent component analysis (ICA). The purpose of an ICA network is to calculate and extract independent components from speech segment by training. Meanwhile, the weight matrix holds the basic function coefficients from the speech segment. One assumption of ICA is that the observation is the linear combination of the independent components [16]. The error rate was 5% at most.

Our proposed method uses both statistical and spectral analysis for extracting all the possible features. Subsequently it selects useful features via a metaheuristic search. The qualified features are then used to reduce the vector dimensionality of training instances for building a classification model. The features from the temporal domain contain richer statistical information than only local maxima and local minima. Our method rides on the observed current trend of fusing information from both time and frequency domains. The merit is that a nonlinear relationship is represented by the spectrum of a spectrum, so only the useful features from the frequency domain in addition to other strong statistical features from the time-domain are encoded into the multidimensional vector which of course is limited in space. Besides, residual and volatility are introduced and embedded into voice classification to produce superior classification result.

### 2.2. Data Mining Algorithms for Voice Classification

Some recent research tapped on the power of data mining algorithms for performing voice classification in various applications. For instance, a new method is proposed by the research team of Lee et al. [17], for prescribing personalized medicine using vocal and facial features. It is a constitution diagnostic method based solely on the individual's physical characteristics, irrespective of psychological traits, characteristics of clinical medicine, and genetic factors. They used Support Vector Machine (SVM) on a software package called LIBLINEAR (L2-loss SVM dual type) for doing voice classification.

As a contribution to telemedicine in home telemonitoring, Maunder et al. [18] investigated the possibility of automatically detecting the sound signatures of activities of daily living of an elderly patient using nonintrusive and reliable methods. A Gaussian mixture model (GMM) classifier was used to differentiate sound activities. Their experiments yielded encouraging results; with recognition accuracies in the range 70% to 100% can be consistently obtained using different microphone-pair positions, under all but the most severe noise conditions.

For biomedical applications, Chenausky et al. made an important contribution in acoustic analysis of Parkinson's disease (PD) speech [19]. The speech of 10 PD patients and 12 normal controls was analyzed for syllable rate and variability, syllable length patterning, vowel fraction, voice-onset time variability, and spirantization. These were normalized by the controls' standard deviation to represent distance from normal and combined into a composite measure. A feedback device that was developed from these findings could be useful to clinicians adjusting deep brain stimulation (DBS) parameters, as a means for ensuring they do not unwittingly choose DBS settings which impair patients' communication.

In our previous work in [1], surveyed several approaches have been studied, such as Artificial Neural Networks (ANN), Support Vector Machines (SVMs), Hidden Markov Models (HMMs), and Gaussian Mixture Models (GMMs). They have been used for training up a classification model with predefined voice samples for voice recognition. A summary of the techniques by which majority of research works used was shown in [1]. In particular, an approach by using unsupervised clustering was described in [1], where priori labeled samples are not required, and the characteristic groupings will be dedicated by the samples themselves. Voiceprints who share similar features will be placed into distinctive groups that represent some labels about the speakers. Subsequently a decision tree (classifier) can be built after studying and confirming the characteristic groups.

Above all the methods a forementioned, encoding techniques from the frequency domains are used as sole features for modeling the voice samples. A single classification algorithm was used specifically for conducting the validation experiment in the literature. In this paper, we advocate combining features from both time and frequency domains, for a throughout coverage of all the voice data characteristics. Then feature selection is used to reduce the dimensionality of the training samples. This way, a minimum subset of relevant features is ensured, and they could be applied into most types of classification models without any limit of a specific type. 

## 3. Proposed Method in Constructing a Voice Classification Model

**Figure 1:**
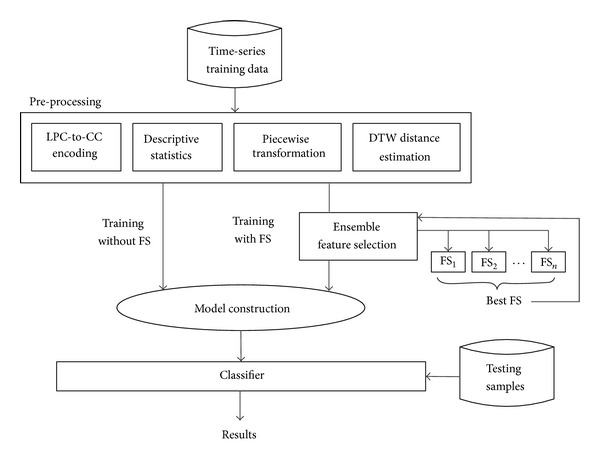
Preprocessing methodology as a part of the classification model learning process.

The SFX preprocessing methodology that is adopted in our research is efficient. Its main merit lies in its ability to transform voice data from one-dimensional to multidimensional features. The SFX technique could possibly fit into a standard data mining process, like the one shown in Figure 1. The training dataset in a form of time-series get converted to multidimensional vectors via the preprocessing process, ready to be used for training a classification model. Given a large dimensionality, ensemble feature selection could be applied over the converted multidimensional vectors for refining the accuracy by retaining only some selected relevant features. In our case, a metaheuristic search method seems to perform very well given its efficient stochastic optimization. Its operational nature is dynamic, suitable for choosing features on the fly, considering that voice data could be potentially continuous. 

**Figure 2:**
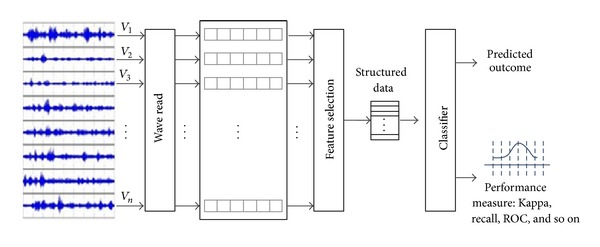
The overall process about SFX.

**Figure 3:**
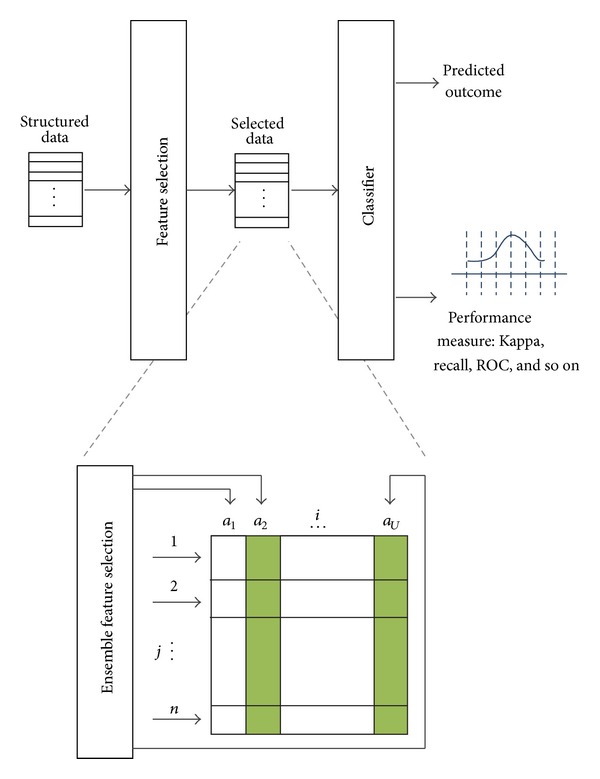
The detailed illustration about SFX with Ensemble FS.

The model construction process is just a standard classification model learning in data mining; for example, a decision tree is built by creating decision paths that map the conditions of the attribute values, as seen from the training samples, to the predicted classes. Once a classifier is trained by processing through the whole training dataset, it is ready to classify new unseen testing samples, and its performance can be measured. The feature selection process is generalized enough to be an ensemble where the winner takes all. During calibration, several feature selection algorithms are put into test, and the best performing one in our case is Feature Selection with Wolf Search Algorithm (FS-WSA) [20]. The other unique contribution by this paper is the extraction of features from the time-series via piece-wise transformation, in addition to the metaheuristic feature selection algorithm. The main difference between our innovation and the others is highlighted in red in Figure 1. We zoom into the details of preprocessing describing the operational flow from data perspective in Figures 2 and 3, respectively, for SFX with and without FS.

In a nutshell, the preprocessing methodology SFX is a way of transforming a two-dimensional time-series (amplitude versus time) into a multidimensional feature vector that has all the essential attributes sufficient to characterize the original time-series voice data. Information is taken from two domains, frequency and time, based on the original time-series. Thus there are two groups of preprocessing techniques being used here, namely, LPC-to-CC encoding (from the frequency domain), Descriptive Statistics of both whole and piecewise, and Dynamic Time Wrap (from the time domain). It is believed that having features obtained from both domains would yield an improved accuracy from the trained classification model due to thorough consideration of the characteristics, hence the representative features, from both domains.

Effectively the preprocessing methodology SFX transforms a matrix of original time-series to a set of training instances which have specific attribute values for building a classification model. Assume *V* (shown in Figure 2 after the wave read process) is an archive of time-series, with each row containing a *j*th time-series *v*_*j*_, and *v*_*j*_ is an ordered sequence of variables *x*_*j*_(*t*) such that *v*_*j*_ = *x*_*j*_(*t*) = (*x*_1_,*x*_2_,…,*x*_*m*_)_*j*_ where 1 ≤ *t* ≤ *m* is the length of the time-series over different time points and 1 ≤ *j* ≤ *n* is the number of instances in the data archive *V*. 

*V* is then to be transformed to a structured training dataset *S* in which each row is an instance *s* that is defined by a finite number of attributes *u*, such that *s*(*j*) = (*a*_1_, *a*_2_,…, *a*_*u*_, *Y*_*j*_) where 1 ≤ *j* ≤ *n* and 1 ≤ *i* ≤ *u*. *a*_*i*_ is the *i*th attribute in *s*(*j*), *Y* is a vector of known target values of *S*; thus *Y*_*j*_ is the *j*th target value to which the attribute values of *s*(*j*) are able to map. The target labels are assumed to be known *a*-priori in *V* (supervised learning), and their values are just carried over from *V* to *S*, instance for instance, by the same order of *j*.

The attributes *a*_1_ ⋯ *a*_*u*_, however, are obtained from the dual time-frequency domains which can be briefly grouped as *s*(*j*) = [(*a*_1_,*a*_2_,…,*a*_*uf*_)_freq_, (*a*_1_,*a*_2_,…,*a*_*ut*_)_time_] where the instance *s*(*j*) is made of two components that are derived from frequency and time domains, respectively. From the frequency domain alone *uf* attributes are extracted, *ut* attributes taken from the time domain, and *u* = *uf* + *ut*.

### 3.1. Feature Extraction from the Frequency Domain

Linear Prediction Coefficients to Cepstral the Coefficients, or Linear Prediction Coding to Cepstrum Coefficients (LPC-to-CC) is selected as the main feature extraction method from the frequency domain in our case. The common production process of human voice contains the following steps of voice generation: the lungs expel air up, acting as the initial step of voice production. Then the air goes into the trachea, passing through the larynx. The larynx is a box-like organ and has two membranes named vocal folds. The voice is actually produced by the vibration of those vocal folds [21]. The acoustic theory of voice production assumes the voice production processes to be a linear system. The output of a linear system is produced based on the linear combination of its previous outputs and current and previous inputs [22]. It is the reason that LPC is chosen here for the purpose of encoding the voice data.

Linear prediction calculates future values of a signal in discrete time format based on a linear function of previous samples. It is always called linear prediction coding, which is a common tool widely used in speech processing for representing the spectral envelope of a signal in compressed form [23].

**Figure 4:**
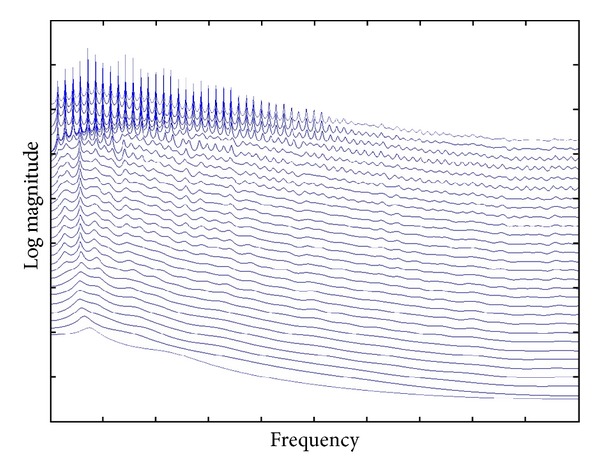
A sample time-series voice data represented in LPC coefficients.

The original time-series voice data *s* is windowed by multiplying a windowing sequence *w*(*n*) via a hamming method, such that *x*(*n*) = *s*(*n*) ⊗ *w*(*n*) where *n* is the window size. It predicts the next values of points as a linear combination of previous points' values. The predicted points with a *p*th order of prediction are as follows:

(1) $$\begin{array}{c} \hat{x}\left(n\right)=\overset{p}{\underset{i=1}{\sum }}a_{i}\cdot x\left(n-i\right), \end{array}$$

where *a*_*i*_ is linear predictor coefficients of the *i*th order.  Figure 4 shows a sample of the predictor coefficients.

The problem of value setting of prediction order *p* determines the characteristics of the vocal filter. If *p* is too low, then key areas of resonance will disappear; if *p* is too high, then characteristics of source are missed. Two complex conjugate poles are needed for characterizing correct formants. Thus, in the signal bandwidth, *p* should be two times of formants number. Suppose *f*_*s*_ is the signal's sampling frequency, and *p* is usually determined as follows:

(2) $$\begin{array}{c} p=\frac{f_{s}}{1000}+\gamma , \end{array}$$

where *γ* is the compensation for glottal roll-off and predictor flexibility, which is normally set to be 2 or 3 [24]. The sampling frequency is usually 10 kHz, so the value of *p* is approximately 12 to 13.

The prediction error generated by this estimate method is the difference between the actual and the predicted values:

(3) $$\begin{array}{c} e\left(n\right)=x\left(n\right)-\hat{x}\left(n\right)=x\left(n\right)-\overset{p}{\underset{i=1}{\sum }}a_{i}\cdot x\left(n-i\right), \end{array}$$

and we define the error metric for the multidimensional signals as

(4) $$\begin{array}{c} e\left(n\right)=||x\left(n\right)-\hat{x}\left(n\right)||=\sqrt{\overset{\infty }{\underset{n=-\infty }{\sum }}{\left[x\left(n\right)-\overset{p}{\underset{i=1}{\sum }}a_{i}\cdot x\left(n-i\right)\right]}^{2}}. \end{array}$$

The expected value of the squared error *E*[*e* ^2^(*n*)] is minimized, yielding the following equation:

(5) $$\begin{array}{c} R_{ss}\left(j\right)=\overset{p}{\underset{i=1}{\sum }}a_{i}\cdot R_{ss}\left(j-i\right)=\overset{|S|-1}{\underset{n=1}{\sum }}x\left(n\right)\cdot x\left(n-i\right), \end{array}$$

where *R*_*ss*_(*j*) is the autocorrelation sequence of signal *x*(*n*).

The autocorrelation sequence can then be represented as a matrix in the format of *R* · *A* = −*r* where *r* is a vector that contains elements of *R*(*x*), and *A* is the vector of predictor coefficients that holds *a*(*y*), for *x*, *y* ∈ [1, *p*]. *R* is known as a Toeplitz Matrix with the size of *p*∗*p* from which the predictor coefficients can be calculated by inverting the matrix *R*, *A* = −*R* ^−1^ *r*. Then the predictor coefficients *A* = [*a*(1), *a*(2),…, *a*(*p*)] can be used to derive the cepstrum coefficients, *c*(*m*), for *m* ∈ [1, *p*], which are the required output of LPC-to-CC. The cepstrum is defined as the inverse DFT of the log⁡⁡ magnitude of the DFT of a signal:

(6) $$\begin{array}{c} c\left(n\right)=F^{-1}\left[\log\left|F\left\{x\left(n\right)\right\}\right|\right], \end{array}$$

where *F* is discrete Fourier transform and *F* ^−1^ is inverted discrete Fourier transform.

When a windowed frame is applied on voice data *y*[*n*], the cepstrum is

(7) $$\begin{array}{c} c\left(n\right)=\overset{N-1}{\underset{n=0}{\sum }}\log\left(\left|\overset{N-1}{\underset{n=0}{\sum }}x\left(n\right)e^{-j(2\pi /N)kn}\right|\right)e^{j(2\pi /N)kn}. \end{array}$$

**Figure 5:**
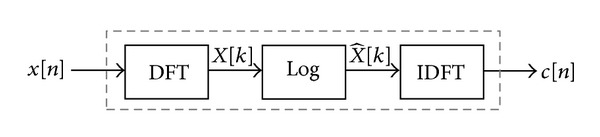
Cepstral Coefficients computation steps.

The transformation steps are shown clearly in Figure 5.

The cepstrum has a lot of advantages such as orthogonality, compactness, and source-filter separation; meanwhile the LPC coefficients are much more susceptible to the precision of numerical numbers, which are less robust than cepstrum coefficients [25]. Thus it is often desirable to transform LPC {*a*_*n*_} into CC {*c*_*n*_}:

(8) $$\begin{array}{c} c_{n}=\{\begin{array}{cc} \ln G & n=0,\\ a_{n}+\frac{1}{n}\overset{n-1}{\underset{k=1}{\sum }}kc_{k}a_{n-k} & 1<n\leq p. \end{array} \end{array}$$

Above all, the transformation converts the original time-series *x*_*j*_(*t*) = (*x*_1_, *x*_2_,…, *x*_*m*_)_*j*_ to a linear prediction coefficient vector defined by (*a*_0_, *a*_1_, *a*_2_,…, *a*_12_)_*j*_ and then converts this vector to a cepstrum coefficient vector defined by (*c*_0_, *c*_1_, *c*_2_,…, *c*_10_)_*j*_. The cepstrum coefficient vector is ready to form a part of the descriptive features, as (*a*_1_, *a*_2_, *a*_*uf*_)_freq_ where *uf* = 10.

### 3.2. Feature Extraction from the Time Domain

Here we have a feature set (*a*_1_,*a*_2_,…,*a*_*ut*_)_time_ that is characterized by a collection of attribute extracted from the time-series of the voice raw data with respect to the time domain. The statistical attribute extraction method has been commonly used by many researchers in the area of digital signal processing, biosignal analysis, and so forth.

#### 3.2.1. Descriptive Statistics

The extracted statistical features include the following statistics: Mean, Standard Deviation, 1st Quartile, 2nd Quartile, 3rd Quartile, Kurtosis, Interquartile Range, Skewness, RSS (residual sum of squares), Standard Deviation of Residuals, Mean Value of Volatilities, and Standard Deviation of Volatilities. Suppose *X*(*t*) is a raw voice data with *N* sampling points, *R*(*t*) is the residual array, and *V*(*t*) is the volatility array.

*Mean*:

(9) $$\begin{array}{c} \overset{-}{X}=\frac{1}{N}\overset{N}{\underset{t=1}{\sum }}X_{t}. \end{array}$$

*Standard deviation*:

(10) $$\begin{array}{c} \sigma =\sqrt{\frac{1}{N}\overset{N}{\underset{t=1}{\sum }}{\left(X_{t}-\overset{-}{X}\right)}^{2}}. \end{array}$$

**Figure 6:**
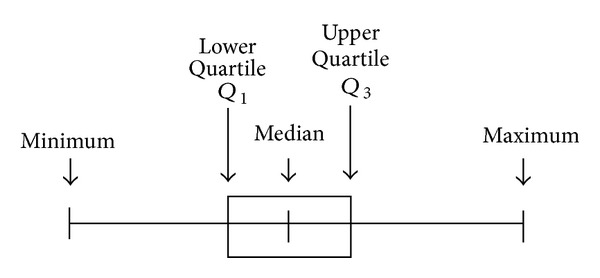
Quartile.

*Quartiles*: (see Figure 6).

*Kurtosis*:

(11) $$\begin{array}{c} K=\frac{\sum_{t=1}^{N}{\left(X_{t}-\overset{-}{X}\right)}^{4}}{\left(N-1\right)\sigma^{4}}. \end{array}$$

A standard normal distribution has the Kurtosis value of three. As the result, the next definition of kurtosis is widely used and it is often known as excess kurtosis:

(12) $$\begin{array}{c} K=\frac{\sum_{t=1}^{N}{\left(X_{t}-\overset{-}{X}\right)}^{4}}{\left(N-1\right)\sigma^{4}}-3. \end{array}$$

*Interquartile range*:

(13) $$\begin{array}{c} \text{IQR}=Q3-Q1. \end{array}$$

*Skewness*:

(14) $$\begin{array}{c} S=\frac{\sum_{t=1}^{N}{\left(X_{t}-\overset{-}{X}\right)}^{3}}{\left(N-1\right)\sigma^{3}}. \end{array}$$

In the statistical analysis of the time-series data, Autoregressive Moving Average models (ARMA) describes a stationary stochastic process based on two polynomials, one for the Auto-regression (AR) and the other for Moving Average (MA) [26]. With the parameter settings this model is usually notated as ARMA(*p*, *q*) where *p* is the order of the AR part and *q* is the order of the MA part.

Now we introduce another model for characterizing and modeling observed time-series: autoregressive conditional heteroskedasticity (ARCH) model. So that in the model, at any time point in this sequence, it will have a characteristic variance.

If an ARMA model is supposed for the build of error variance, then the model is a Generalized Autoregressive Conditional Heteroskedasticity (GARCH) model [27]. With the parameter settings this model is usually referred to as the GARCH(*p*, *q*) where *p* is the order of the GARCH terms *σ* ^2^ and *q* is the order of the ARCH terms *ϵ* ^2^:

(15) σt2=α0+α1ϵt−12+⋯+αqϵt−q2+β1σt−12+⋯+βpσt−p2=α0+∑i=1qαiϵt−i2+∑i=1pβiσt−i2.

We set the parameters of GARCH model with standard values such as the following.

 Distribution = “Gaussian”;
 variance Model = “GARCH”;
 
*p* (model order of GARCH(*p*, *q*)) = “1”;
 
*q* (model order of GARCH(*p*, *q*)) = “1”;
 
*r* (autoregressive model order of an ARMA(*r*, *m*) model) = “1”.

*RSS*:

(16) $$\begin{array}{c} \text{RSS}=\overset{N}{\underset{t=1}{\sum }}{\left(X_{t}-\hat{X_{t}}\right)}^{2}. \end{array}$$

*Standard deviation of residuals*:

(17) $$\begin{array}{c} \text{resstd}=\sqrt{\frac{1}{N}\overset{N}{\underset{t=1}{\sum }}{\left(R_{t}-\overset{-}{R}\right)}^{2}}. \end{array}$$

*Mean value of volatilities*:

(18) $$\begin{array}{c} \text{volmean}=\frac{1}{N}\overset{N}{\underset{t=1}{\sum }}V_{t}. \end{array}$$

*Standard deviation of volatilities*:

(19) $$\begin{array}{c} \text{volstd}=\sqrt{\frac{1}{N}\overset{N}{\underset{t=1}{\sum }}{\left(V_{t}-\overset{-}{V}\right)}^{2}}. \end{array}$$

#### 3.2.2. Dynamic Time Warping Distance

Though descriptive statistics may give us the overall summary of time-series data and characterize a general shape of time-series data, they may not be able to capture the precise trend movements which are also known as the patterns of evolving lines. In particular we are interested in distinguishing the time-series which belong to one specific class from those that belong to another class. The difference of trend movements can be estimated by a technique called Dynamic Time Warping.

Dynamic Time Warping (DTW) is an algorithm for measuring similarity between two time-series in the situation that both have similar shapes but they vary in time step or speed rate. DTW has been applied to many data objects like video, voice, audio, and graphics. Actually, DTW can explain and deal with any ordered set of data points by the format of linear combination [28].

In theory, DTW is most suitable for voice wave patterns because exact matching for such patterns often may not occur, and voice patterns may vary slightly in the time domain. DTW finds an optimal match between two sequences that allows for compressed sections of the sequences. In other words it allows some flexibility for matching two sequences that may vary slightly in speed or time. The sequences are “warped” nonlinearly in the time dimension to determine a measure of their similarity independent of certain nonlinear variations in the time dimension. Particularly suitable DTW is for matching sequences that may have missing information or various lengths, on condition that the sequences are long enough for matching.

Suppose that *x*_*j*_(*t*), 1 ≤ *j* ≤ *n* represents an instance in time-series archive *X*, the number of instances in *X* is *n*. *c*_*i*_, 1 ≤ *i* ≤ *m* means each class label to which every instance belongs, where *m* is the number of class labels. *Y*_*j*_, 1 ≤ *j* ≤ *n* is the *j*th target value to which the attribute values of *x*_*j*_(*t*) are able to map. *N*_*i*_, 1 ≤ *i* ≤ *m* is the number of target values in each class *c*_*i*_. For any *x*(*t*) in time-series archive *X*, the DTW distance of *x*(*t*) to its own class *c*_*i*_ is defined as

(20) $$\begin{array}{c} \mathrm{dist}=\frac{1}{N_{i}}\overset{N_{i}-1}{\underset{r=1}{\sum }}d_{ir}. \end{array}$$

Note that the count upper limit is *N*_*i*_ − 1 because the DTW distance between *x*(*t*) and itself is 0 by the definition (they have the exactly same shape). The DTW distance of *x*(*t*) to another class *c*_*j*_ to which it does not belong is

(21) $$\begin{array}{c} \mathrm{dist}=\frac{1}{N_{j}}\overset{N_{j}}{\underset{r=1}{\sum }}d_{ir}. \end{array}$$

**Figure 7:**
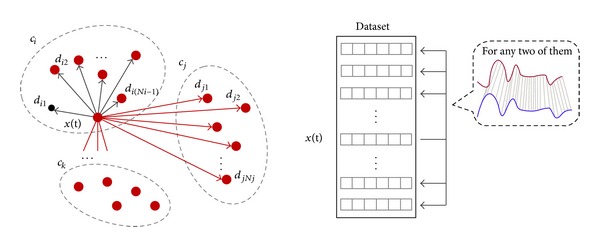
Illustration of DTW calculation.

So the number of distance attributes equals the number of *c*_*i*_, that is, how many classes in total. These distance attributes compose a member of features in (*a*_1_,*a*_2_,…,*a*_*ut*_)_time_, which represents the extracted features of a whole time-series raw data in time domain.  Figure 7 visually illustrates this concept of distance in DTW computation.

#### 3.2.3. Piecewise Transformation

So far along the time-domain, statistics are extracted from the whole piece of the time-series as well as the similarity in terms of distance between the test time-series and the mean of its peer group. For a finer level of information, a piecewise transformation is applied which is called Piecewise Linear Function (PLF). A continuous time-series is converted into a collection of linear segments when PLF is applied on it. The purpose of this compressed expression method is to approximate a polynomial curve into a vector of finite *n*-dimensional Euclidean space that consists of quantitative values.

This is the key part of the research work because it contains our new contribution. Inspired by the financial analysis of stock market, residual and volatility are firstly imported in the application field of voice classification. Like historical volatility for one or more stocks over some specified trading days, we also believe that certain patterns of someone's speech are involved in residual and volatility.

Each sentence is read by *wavread* function in MATHLAB into a one dimension array as illustrated in Figure 2. The starting and ending points of every time-series data are just the same as the beginning and ending points of each array, which means that all information is used without any redundancy. The depth of segmentation *n* can be selected arbitrarily but sufficiently by the user. In our experiments, the average length of a sentence is ten words, and each word has a peak correspondingly. The mean length of the sampled time-series array is 100 k points. Without compromising the resolution and the complexity of feature space, we choose *n* to be 20, thus we can cut a peak into two parts which represents up and down gradients. Then the continuous time-series voice data is partitioned equally into 20 pieces.

**Figure 8:**
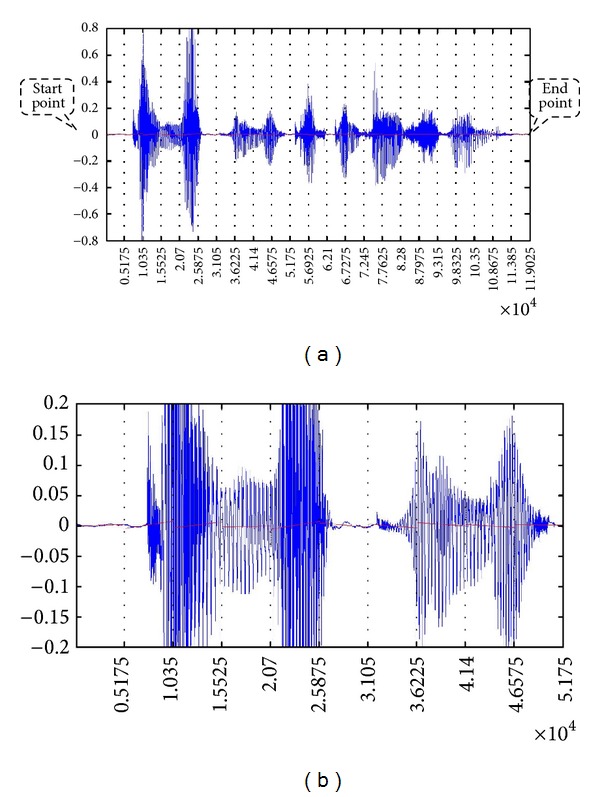
(a) An example of sampled time-series voice data and its partition. (b) The amplified view of piecewise linear regression (partly).

In our experiment, we try to keep the length of every spoken sentence the same, being almost 10 k points after sampling. The number of segmentations is also 20, so each piece maintains at nearly 5 k sampling points. For each segment of the time-series, certain statistics that describe the trend and dynamics of the movement are extracted into the feature vector, that is, (*a*_1_, *a*_2_,…, *a*_*ut*_)_time_. An example of the time-series segmentation in normal and stretched view is shown in Figure 8.

Using this piecewise method, the features that are being extracted are statistics of each partition of the time-series.  Table 1 shows a list of all statistics that can potentially be harvested from 20 partitions of a particular time-series. The definitions of the statistics parameters then follow.

For each segment *s*_*i*_(*t*), 1 ≤ *i* ≤ 20, *n* is the number of points each segment contains, that is, *n* = |*s*_*i*_(*t*)|.

*Gradient of s*_*i*_(*t*):

(22) $$\begin{array}{c} {\text{grad}}_{i}=\beta_{i}, \end{array}$$

where *β*_*i*_ is

(23) $$\begin{array}{c} s_{i}=\beta_{i}t+\alpha_{i}+\varepsilon_{i}. \end{array}$$

*RSS of s*_*i*_(*t*):

(24) $$\begin{array}{c} {\text{RSS}}_{i}=\overset{n}{\underset{t=1}{\sum }}{\left(s_{it}-\hat{s_{it}}\right)}^{2}. \end{array}$$

*Standard deviation of residuals of s*_*i*_(*t*):

(25) $$\begin{array}{c} {\text{resstd}}_{i}=\sqrt{\frac{1}{n}\overset{n}{\underset{t=1}{\sum }}{\left(\varepsilon_{it}-{\overset{-}{\varepsilon }}_{i}\right)}^{2}}. \end{array}$$

*Mean value of volatilities of s*_*i*_(*t*):

(26) $$\begin{array}{c} {\text{volmean}}_{i}=\frac{1}{n}\overset{n}{\underset{t=1}{\sum }}V_{it}. \end{array}$$

*Standard deviation of volatilities of s*_*i*_(*t*):

(27) $$\begin{array}{c} {\text{volstd}}_{i}=\sqrt{\frac{1}{n}\overset{n}{\underset{t=1}{\sum }}{\left(V_{it}-\overset{-}{V_{i}}\right)}^{2}}. \end{array}$$

The model for residual and volatility is also selected as GARCH model, where the parameters of GARCH model are configured the same as previously: mentioned Distribution = “Gaussian”; Variance Model = “GARCH”; *p* (model order of GARCH(*p*, *q*)) = “1”; *q* (model order of GARCH(*p*, *q*)) = “1”; *r* (autoregressive model order of an ARMA(*r*, *m*) model) = “1”.

**Figure 9:**
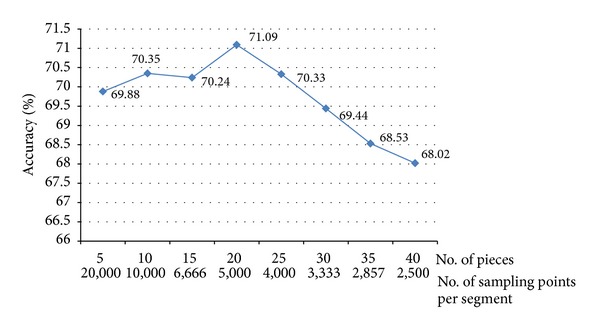
Calibration curve for segmentation selection.

A calibration test is used to determine the optimal choice of the length of each piece (interval) such that the highest classification accuracy can be obtained. Different numbers of intervals have been tried continually for piecewise transformation, extracting the corresponding attributes and running the classifiers. As the results shown in Figure 9, it was found that using 20 segments of each length yields the highest classification accuracy. The test was done preliminarily without FS and the results are averaged over all parameters.

## 4. Experiment

In order to compare the effectiveness of the proposed time-series preprocessing method with the other existing methods, we test them on four different voice/speech datasets using nearly twenty popular and traditional classification algorithms in data mining.

### 4.1. Data Description

Four representative types of voice data are tested by the simulation experiments; they are Female and Male (FM) Dataset, Emotional Speech (ES) Dataset, Speaker Identification (SI) Dataset, and Language Recognition (LR) Dataset.

#### 4.1.1. Data Sources

*FM*. The FM dataset is downloaded from School of Information Technology and Electrical Engineering (ITEE), University of Queensland, Australia, called VidTIMIT Audio-Video Dataset [29]. The dataset is made up of audio recordings of recited short sentences from 43 volunteers, among which 19 are females and 24 are males. It is from the test section of TIMIT corpus that all those sentences were selected. 10 sentences for every speaker. The first two sentences are all the same for each speaker, with the remaining eight that differ according to every individual. Here only the audio data is concerned and video data is discarded. 

*ES*. The ES dataset comes from the database of German emotional speech, developed at the Technical University, Institute for Speech and Communication, Department of Communication Science, Berlin, with Professor Sendlmeier. It was funded by the German Research Association DFG (research project SE 462/3-1) [30]. The aim of the database is to examine acoustical correlates of emotional speech. It is comprised of seven basic emotions (anger, happiness, sadness, fear, disgust, boredom, and neutral) and only four major emotions are taken for the purpose of simplification. Ten professional native German actors with balance gender distribution (5 for each) produced these emotional speeches, which containing 10 sentences with 5 short sentences and 5 longer ones. 

*SI*. The SI dataset is taken from the PDA speech database, owned by Yasunari Obuchi in March 2003, Carnegie Mellon University (CMU). The recording was done by CMU students and staff [31]. There recording was done by using one PDA with four small microphones mounted around and one big microphone in the record room. The type of that big microphone was an Optimus Nova 80 close-talk microphone. The type of small ones was Panasonic WM-55DC2 and they were mounted using a mock-up shown below. There are 16 speakers and each read about 50 sentences. 

*LR*. The LR dataset is generated through an approach called speech synthesis. The speech synthesizer software used here is Microsoft Text-to-Speech engine with many expansion packages [32]. Sentences of English, Cantonese, and Mandarin were widely selected from the area of frequently used daily conversations, daily news, educational reports, stories, scientific articles, ancient proses, poems and poetries, and so forth.

#### 4.1.2. Data Formats

The voice data is in the format of two-dimensional time-series, with an amplitude value in sound that varies over time; examples are given in Figures 8(a) and 8(b). The sampling rate or frequency of wave read process is 10 kHz. Group distributions of distinctive datasets are given in Table 2. The FM dataset has only two classes, which is the simplest classification task in data mining. The rest of datasets contain more than two classes that make the classification task more difficult. The numbers of attributes or features for every dataset and instances for training and testing are listed in Table 3.

#### 4.1.3. Data Visualization

**Figure 10:**
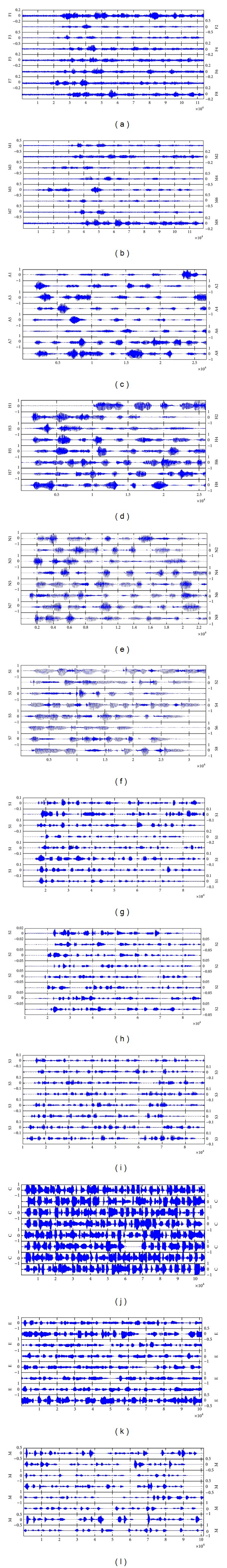
(a) Visualization of FM dataset that belongs to the “Female” group. (b) Visualization of FM dataset that belongs to the “Male” group. (c) Visualization of ES dataset that belongs to the “Anger” group. (d) Visualization of ES dataset that belongs to the “Happiness” group. (e) Visualization of ES dataset that belongs to the “Neutral” group. (f) Visualization of ES dataset that belongs to the “Sadness” group. (g) Visualization of SI dataset that belongs to the “Speaker 1” group. (h) Visualization of SI dataset that belongs to the “Speaker 2” group. (i) Visualization of SI dataset that belongs to the “Speaker 3” group. (j) Visualization of LR dataset that belongs to the “Cantonese” group. (k) Visualization of LR dataset that belongs to the “English” group. (l) Visualization of LR dataset that belongs to the “Mandarin” group.

Visualization of parts of each group of the datasets, FM, ES, SI, and LR is displayed in Figures 10(a) to 10(l). Inspecting by just naked eyes, one can see some distinctive differences between the waveforms of different classes.

**Figure 11:**
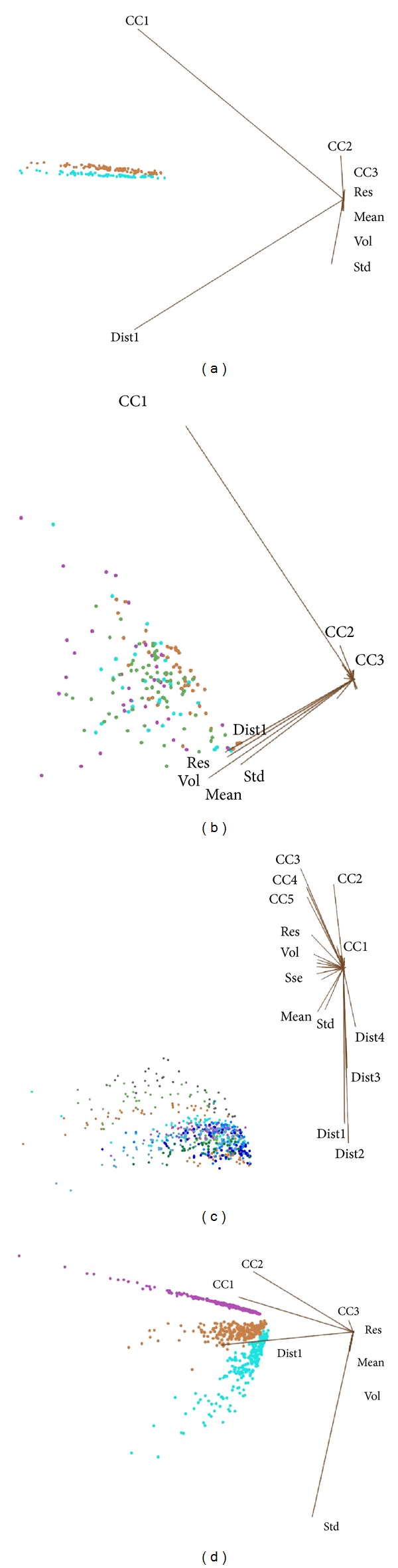
(a) MD visualization of FM. (b) MD visualization of ES. (c) MD visualization of SI. (d) MD visualization of LR.

Multidimensional (MD) visualization of each group of those datasets is shown in Figures 11(a) to 11(b). Again, by just visual inspection, it can be observed that the voice data between different classes are apparently distinctive in the FM group and in the LR group. Common sense tells us that female speakers and male speakers have distinguishing vocal tones. Speeches of different languages also can be differentiated easily, as each language has its unique vowels and phonics. In contrast, the voice data of 16 unique speakers have certain overlaps in their feature values; this implies that some speakers share similar voices which are not something very uncommon in real life. The voice data in the emotion groups are highly mixed together by the feature values. That shows the potential computational difficulty in classification between voices of different emotions.

#### 4.1.4. Algorithms Used in Comparison

Our experiments are performed by using popular and standard classification algorithms (with their default parameters applied) over the four sets of the above-mentioned voice data that are being handled by four preprocessing methods. A total of 20 classification algorithms are being used. The justification is that we try to test the generality of our voice classification model without being attached to any specific classification algorithm. In other words, the design of the voice classification model should be generic enough, and its efficacy should be independent from the choice of classifier. While the focus of the voice classification model is centered at the preprocessing steps which leverage the features from both time and frequency domains followed by feature selection for reducing the feature space dimension, classification algorithms can become flexible plug-and-play in our model design. The standard classification algorithms used in our experiments are well known in data mining research community as well as available in Weka (http://www.cs.waikato.ac.nz/ml/weka/), and they are listed in Table 4.

The four preprocessing methods used for comparison are as follows.

*LPC-to-CC*. Only the cepstrum coefficients are used as the encoding result of time-series voice data. Meanwhile, the LPC coefficients are ignored in final attributes set. 

*Wavelet*. Only the 50-largest Harr wavelet coefficients are taken as converting the sequence from time domain to frequency domain. The number of decomposition level of Harr wavelet transform is 3. 

*SFX*. Statistical Feature Extraction (SFX) converts the time-series voice data to a whole set of attributes with both frequency and time domains, using a collection of feature methods described in Section 3. 

*SFX + FS*. Statistical Feature Extraction + Feature Selection (SFX + FS) is exactly the same as SFX except that the full set of features or attributes were filtered by using different feature reduction methods. Note that it is an ensemble feature selection method, using multiple models to obtain the best performance. Two facts are considered: mean accuracy and time cost. The compensation is made between time and accuracy, which means that we prefer a little bit lower accuracy and more on acceptable time cost. The optimal one was chosen as the final FS method. 

*WSA*. Wolf Search Algorithm (WSA) is a bioinspired heuristic optimization algorithm [33]. It naturally balances scouting the problem space in random groups (breadth) and searching for the solution individually (depth). The pseudocode of WSA is given in Pseudocode 1. 

*Chi-Square*. In statistics, the purpose of chi-square (*χ* ^2^) test is to measure the independence of two events *A* and *B*. From the knowledge of probability and statistics, we know that two events are independent if the probability equation has the following relationships: *P*(*A* | *B*) = *P*(*A*) and *P*(*B* | *A*) = *P*(*B*) or *P*(*AB*) = *P*(*A*)*P*(*B*) equivalently. In feature selection, let occurrence of the term be event *A* and occurrence of the class be event *B*. We then rank values based on the following quantity [34, 35]:

(28) $$\begin{array}{c} \chi^{2}\left(D,t,c\right)=\underset{e_{t}\in \{0,1\}}{\sum }\;\underset{e_{c}\in \{0,1\}}{\sum }\frac{{{(N}_{e_{t}e_{c}}-E_{e_{t}e_{c}})}^{2}}{E_{e_{t}e_{c}}}, \end{array}$$

where *D* is the whole set of observations, *N* is the frequency actually found in *D*, and *E* is the expected one. At the same time, *e*_*t*_ = 1 means that the document contains term *t*, *e*_*t*_ = 0 means that the document does not contain *t*, *e*_*c*_ = 1 means that the document is in class *c*, and *e*_*c*_ = 0 means that the document is not in *c*. 

*CFS*. An essential assumption is made before going directly into the discussion of Correlation Feature Selection (CFS). It is that good feature subsets always have highly corresponding features, whereas there are uncorrelated features among the rest of them [36]. On the basis of that, CFS starts its work and evaluates features. The merit containing *k* features for a specific feature subset *S* is

(29) $$\begin{array}{c} {\text{Merit}}_{S_{k}}=\frac{k\overset{-}{r_{cf}}}{\sqrt{k+k\left(k-1\right)\overset{-}{r_{ff}}}}, \end{array}$$

where $\overset{-}{r_{cf}}$ represents the average value of all *c*-*f* (classification to feature) correlations, and $\overset{-}{r_{ff}}$ is the mean value of all *f*-*f* (feature to feature) correlations. Then CFS is defined as follows:

(30) $$\begin{array}{c} \text{CFS}=\underset{S_{k}}{\max}\frac{r_{cf_{1}}+r_{cf_{2}}+\cdots +r_{cf_{k}}}{\sqrt{k+2\left(r_{f_{1}f_{2}}+\cdots +r_{f_{i}f_{j}}+\cdots +r_{f_{k}f_{1}}\right)}}, \end{array}$$

where *r*_*cf*_*i*__ and *r*_*f*_*i*_*f*_*j*__ variables are correlations just like the aforementioned. 

*MRMR*. Maximum Relevance is normally referred to as subsets of data identified by feature selection which are relevant to the parameters. There often exist relevant but redundant components in those subsets. MRMR, known as Minimum Redundancy Maximum Relevance, however, attempts to detect those redundant subsets, find them out, and delete them. Example application fields of MRMR are but not limited to cancer diagnosis, face detection, autoresponse, and speech recognition.

Suppose *p*(*x*), *p*(*y*), and *p*(*x*, *y*) to be probabilistic density functions of two random variables *x* and *y*; then their mutual information is defined as [37]

(31) $$\begin{array}{c} I\left(x;y\right)=\iint p\left(x,y\right)\log\frac{p\left(x,y\right)}{p\left(x\right)p\left(y\right)}dx\;dy. \end{array}$$

The nature of feature selection in mutual information model is to find a feature set *S* containing *m* features {*x*_*i*_}, which also have the largest dependency on the target class *c*. This is the definition of Max Dependency:

(32) $$\begin{array}{c} \max D\left(S,c\right),\;D=I\left(\left\{x_{i},i=1,2,\ldots ,m\right\};c\right). \end{array}$$

Max-Relevance and Min-Redundancy are

(33) $$\begin{array}{c} \max D\left(S,c\right),\;D=\frac{1}{\left|S\right|}\underset{x_{i}\in S}{\sum }I\left(x_{i};c\right),\\ \min R\left(S\right),\;R=\frac{1}{{\left|S\right|}^{2}}\underset{x_{i},x_{i}\in S}{\sum }I\left(x_{i},x_{j}\right). \end{array}$$

Out of the chosen popular feature selection algorithms that are put into test in the calibration process, we can see that WSA which is a metaheuristic FS algorithm consistently is having superior performance, except for the Speaker Identification dataset which is known for its overlaps in feature values. The testing results are shown in full in Table 5. The computing environment is on a PC workstation, with Windows 7 Enterprise Edition, 64 bits, Intel Core i7 CPU, and 8 GB RAM. 

**Pseudocode 1:**
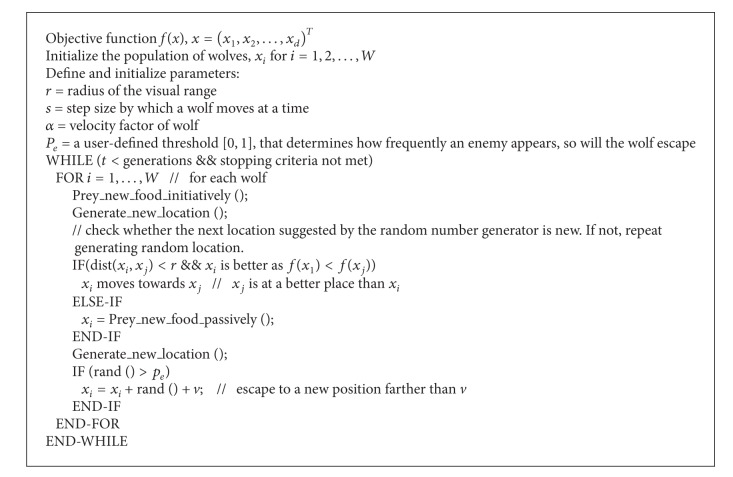
Pseudocode of WSA.

## 5. Results and Analysis

The objective of our experiments is to compare the performance of those four preprocessing methods on four kinds of voice datasets when a collection of data mining classifiers are applied. Our performance evaluation covers four main aspects: (1) accuracy comparison of datasets; (2) accuracy comparison of preprocessing methods; and (3) overall averaged performance comparison.

Twenty popular classification algorithms were used on FM and LR datasets, which is regarded as a representative set of commonly used classifiers. However, the classifier of LibSVM could not be applied on ES and SI due to their formats. Some attribute data contain infinitely small values. Results from some classifiers are not available because of the time limitation: it takes too much time for them to build a classification model when the number of attributes gets very large. As such, LibSVM is excluded from experiments involving ES and SI. NBTree and Conjunctive Rule are excluded from experiments over the dataset SI. For feature selection, the algorithm candidate that yields the highest accuracy is used in the subsequent experiments.

### 5.1. Accuracy Comparison of Datasets

**Figure 12:**
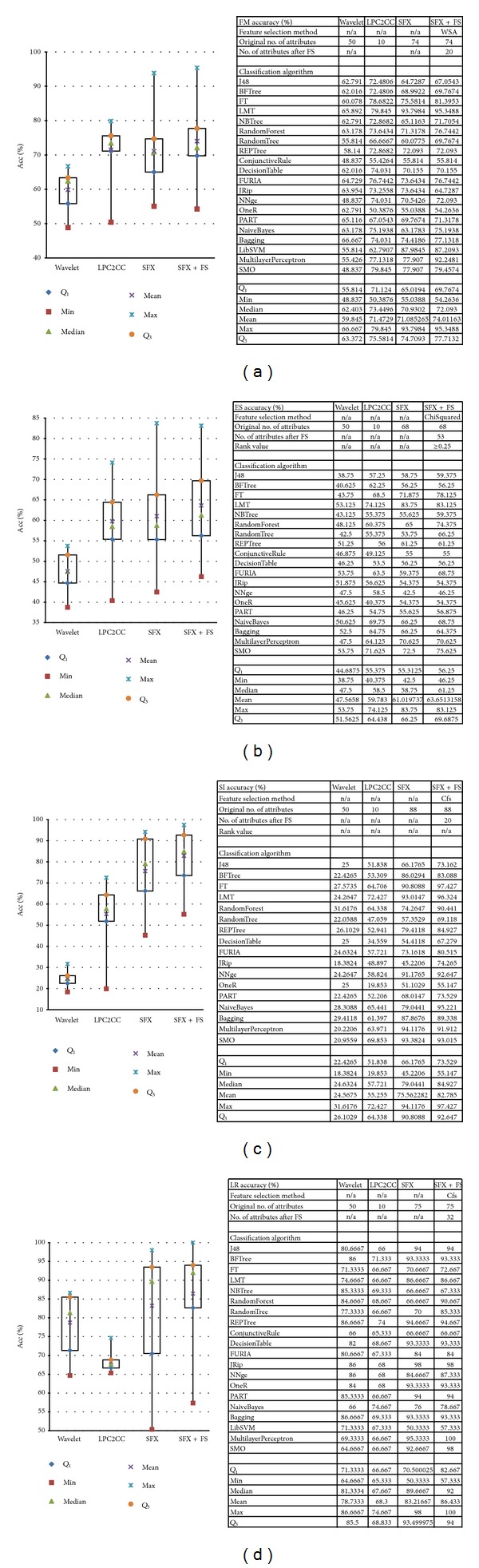
(a) FM boxplot and accuracy table. (b) ES boxplot and accuracy table. (c) SI boxplot and accuracy table. (d) LR boxplot and accuracy table.

The accuracy of the classification result is the most significant criterion for evaluating the performance. It is defined as the percentage of correctly classified instances over the total number of instances. This section shows total accuracies of four preprocessing methods on each voice dataset. Four sets of accuracy results and box plots for different dataset are presented in Figures 12(a) to 12(d).

From the aforementioned figures we find that the first two preprocessing methods, which are wavelet and LPC-to-CC, yielded a relatively nonstationary accuracy result on all four datasets. For LR dataset, wavelet method generated better result than LPC-to-CC. Conversely, LPC-to-CC was better for FM, ES, and SI. Recalling from Section 4.1.1, we know that only the LR dataset is synthetic, which was produced by a Text-to-Speech engine. LPC-to-CC, known as a common voice encoding method, has a problem in obtaining the more realistic components: there are many transition frames that the LPC model fails to sort correctly [38]. Such inaccuracy of the model might be due to annoying artifacts like buzzes and tonal noises. So the performance was relatively worse.

Meanwhile, SFX and SFX + FS showed relatively more stable results than the first two. They really improved the accuracy a lot. By a contrast of SFX and SFX + FS, after feature selection, the main range (*Q*_3_–*Q*_1_) of accuracy distribution became narrower and the accuracy results increased.

**Figure 13:**
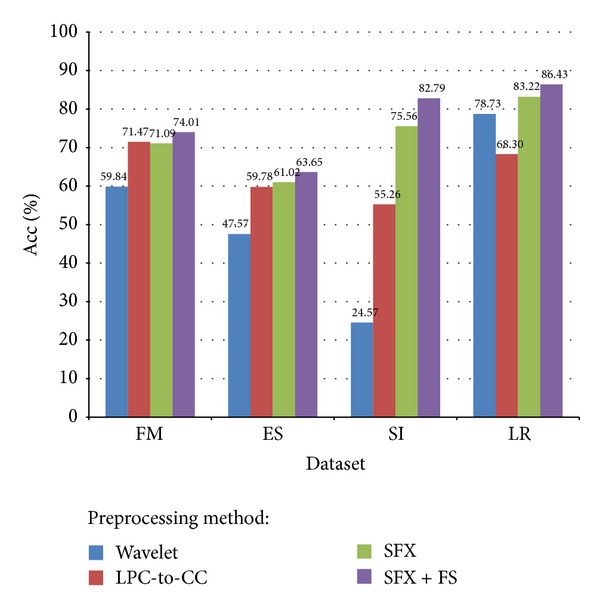
Comparison of average accuracy for different voice datasets and different preprocessing methods.

More evident comparison result is given when the accuracies are averaged out and placed together side by side in a bar chart in Figure 13.

An interesting phenomenon is observed from Figure 13—the accuracy fell a little after SFX compared to LPC-to-CC over FM dataset. However, from the methodology of SFX, we know that cepstral coefficients are involved in the attributes of SFX. This indicates that the classification accuracy may decrease when the number of attributes increases due to the redundancy of those unnecessary features [39]. FM has only binary classes; the performances of the preprocessing methods differ very little compared to those in other datasets that have multiple classes. In particular, SI has 16 different classes; the differences of performance between the preprocessing methods become obvious.

Another considerable fact is also derived from Figure 12 on LR dataset—Wavelet seemed to have a better performance than what LPC-to-CC did. Besides the drawback of LPC encoding method, we can also consider other reasons. The inherent frequency of one's speech is an important acoustic feature for identifying different individuals. Other necessary features may include behavioral patterns (such as voice pitch and speaking style) and human anatomy patterns (like the shape of throat). Remember that the result of LPC-to-CC only contains 10 cepstral coefficients, and the number of target groups to be classified is 16. It contains too few information for correct classification and wavelet provides relatively sufficient features.

Considering the number of classes in each dataset together with the accuracy result, we can find that the accuracy of binary targets classification (FM) is higher than multiple targets classification (ES) and (SI) for the frequency-domain encoding methods. For the time-domain methods like SFX and SFX-FS, good accuracy still can be attained in multiclass classification as in SI where the frequency-domain methods underperform.

Multiclass classification categorizes instances into more than two classes, whereby a hypothesis is constructed to make sure that discriminates can be distinguished between a fixed set of classes. An assumption is made before that, which is closed set and good distribution. If all possible instances belonging to each case fall into one of the classe, and each class contains statistically representative instances, then the performance of classification is good enough. For now, the boundary of every emotion in ES dataset is not clear (which is already shown in Figure 11(b)), so it does not meet the condition of closed set, and the result is worse than FM. For SI and LR, the features of each individual and language are discriminative enough to tell all classes apart, meaning that they are well distributed, so the results are better than FM.

### 5.2. Accuracy Comparison of Preprocessing Methods

**Figure 14:**
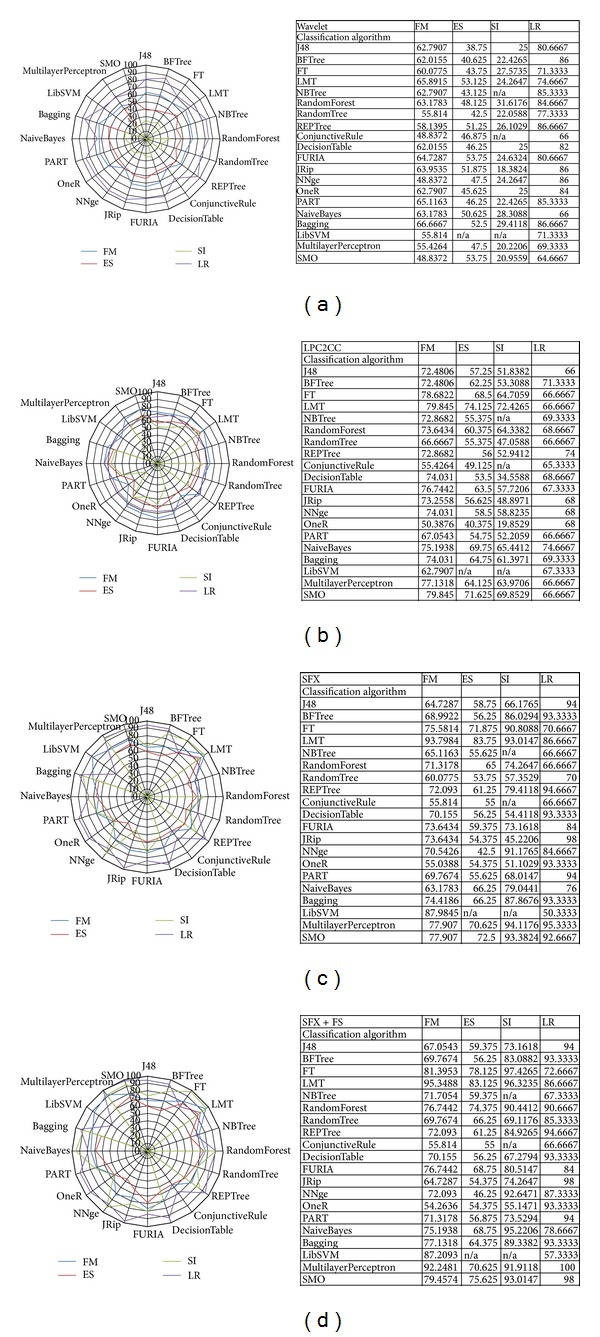
(a) Accuracy comparison of Wavelet preprocessing method. (b) Accuracy comparison of LPC-to-CC preprocessing method. (c) Accuracy comparison of SFX preprocessing method. (d) Accuracy comparison of SFX + FS preprocessing method.

This section shows the accuracies of four datasets when every preprocessing method is applied on them, respectively. Four sets of accuracy results and radar charts by different preprocessing methods are shown in Figures 14(a) to 14(d).

It can be seen that in general the classification algorithms produce consistent results when wavelet and LPC-to-CC preprocessing methods are used. These almost all-rounded accuracy results are displayed in Figures 14(a) and 14(b). Comparatively, SFX and SFX + FS yield a jagged outline for the curves of accuracy results in the radar chart, which can be seen in Figures 14(c) and 14(d). Overall, Wavelet and LPC-to-CC show lower average accuracy than those in SFX and SFX + FS. Some classifiers produce exceptionally perfect accuracy on all the four datasets after statistical feature extraction and feature selection are applied. They are LMT and Multilayer Perceptron.

The classifier model generated from LMT is a single tree with different shapes on basis of various types of training data. If the data type is numeric, then a binary tree will be built with splits on those attributes; if the type is nominal, then a multi-split tree is the consequence. But the same thing is that the leaves are each logistic regression model which is quite capable for analysis of dataset with dependent features and bounded magnitudes of time-series. The algorithm is guaranteed that only relevant attributes are selected [40]. The result is much more intelligible and reasonable than a committee of multiple trees on voice classification. So under such kind of circumstance, LMT offers a better result than other tree classifiers.

Multilayer Perceptron is a standard algorithm for any supervised learning task in data mining. The result is relatively better than any other classifiers, achieving almost 100% accuracy but the time cost is higher and sometimes unacceptable. However, some classifiers produce low accuracy, for instance, Naïve Bayes. Based on Bayes' theorem with strong independence assumptions, Naïve Bayes acts as quite a simple classifier and it gets very widely adopted in many classification situations. But sometimes the relation between any pair of attributes is always dependent and the distribution of features is unknown in advance; thus the performance of such a simple probabilistic classifier is bad and unstable.

### 5.3. Overall Averaged Performance Comparison

For a throughout performance evaluation, performance consideration of other parameters is considered as well; these include Kappa, Precision, Recall, F1, and ROC, which are commonly used in assessing the quality of the classification models in data mining. These performance indicators are briefly described as follows. The performance results pertaining to these indicators are averaged over all the four datasets and all the 20 classification algorithms. They are then shown in Section 5.3.6 together with the comparison of time cost.

#### 5.3.1. Kappa Statistic

**Figure 15:**
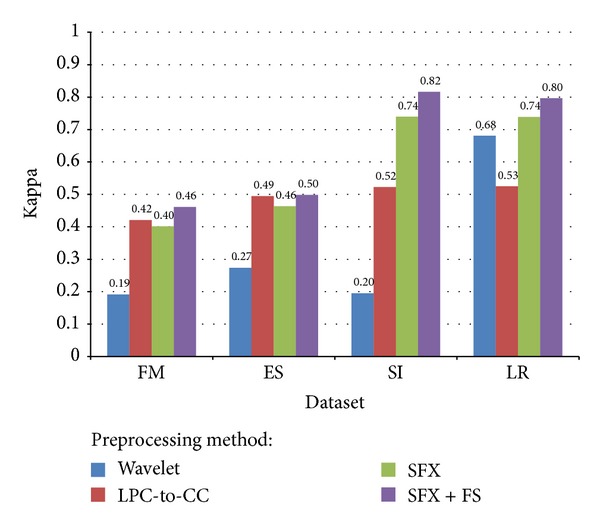
Comparison of average Kappa statistic for different voice datasets and different preprocessing methods.

Kappa statistic is widely used to measure variability between multiple observers. The meaning of Kappa statistic is how often multiobservers agree in terms of their interpretations. When two or more evaluators are checking the same data, Kappa statistic is assessed to show an agreement of evaluators when the same data categories are correctly assigned. As well known, simple agreement just between yes and no is poor because of the property of chance and arbitrary. That is why Kappa statistic is introduced and it is preferred [41]. The definition of Kappa statistic is given as follows:

(34) $$\begin{array}{c} \kappa =\frac{\text{Pr}\left(a\right)-\text{Pr}\left(e\right)}{1-\text{Pr}\left(e\right)}, \end{array}$$

where Pr(*a*) is the relative observed agreement among raters and Pr(*e*) is the hypothetical probability of chance agreement. When the application is classification, the measure of chance between the classification results and the true classes (labeled categorical data class) is assessed by Kappa statistic. It reflects the reliability of the evaluation of our classifier.  Table 6 is the general criterion of evaluating Kappa statistic [42]. A comparison of different voice datasets and different preprocessing methods, in terms of average Kappa statistic, is shown in Figure 15. Wavelet method is relatively unstable in datasets of FM, ES, and SI. The Kappa statistics for LPC-CC method are almost the same across different datasets. SFX without FS, however, underperformed when compared to LPC-CC in FM and ES datasets which are relatively simple. SFX-FS shows its superiority in Kappa statistics in all datasets.

#### 5.3.2. Precision

In pattern recognition and data mining, precision is the fraction of relevantly retrieved instances. In the situation of classifications, the terms positive and negative describe the classifier's prediction results, and the terms true and false refer to whether the prediction results correspond to the fact or not [43]. This is illustrated by Table 7.

Precision is defined as:

(35) $$\begin{array}{c} \text{Precision}=\frac{\text{TP}}{\text{TP}+\text{FP}}. \end{array}$$

**Figure 16:**
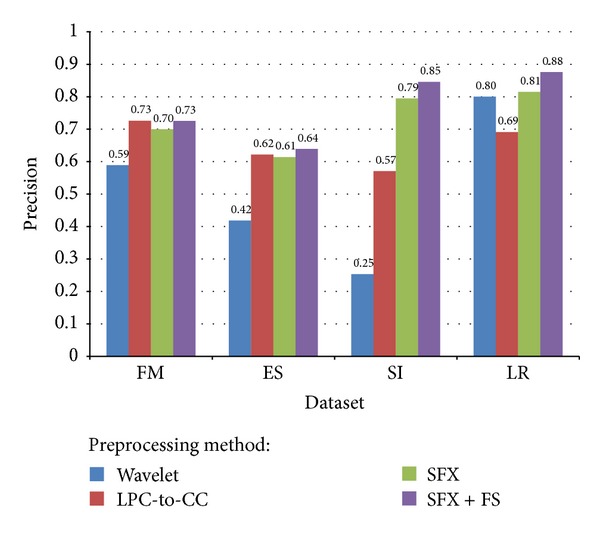
Comparison of average precision for different voice datasets and different preprocessing methods.

Precision is concisely defined as “of all the instances that were classified into a particular class, how many were actually belonged to that class?” In classification task, a perfect precision score for a particular class means that every instance classified into that class does indeed belong to that class (but it says nothing about the number of instances from that class that were not classified correctly). As shown in Figure 16, for example, SFX-FS when applied on LR dataset has the maximum precision score 0.88—that means 88% of the instances that are classified into a particular indeed belong to that class. SFX-FS for SI has precision score 0.85, for ES has only 0.64, and for FM has 0.73. Wavelet method was unacceptable for all datasets except LR, for it has merely 0.59, 0.42, and 0.25 precision scores, respectively. The comparison with respect to precision scores is shown in Figure 16.

#### 5.3.3. Recall

In pattern recognition and data mining, recall is defined as the fraction of relevantly retrieved instances. We can infer that the same part of both precision and recall is relevance, based on which they all make a measurement. Usually, precision and recall scores are not discussed in isolation and the relationship between them is inverse, indicating that one increases and the other decreases. Recall is defined as

(36) $$\begin{array}{c} \text{Recall}=\frac{\text{TP}}{\text{TP}+\text{FN}}. \end{array}$$

**Figure 17:**
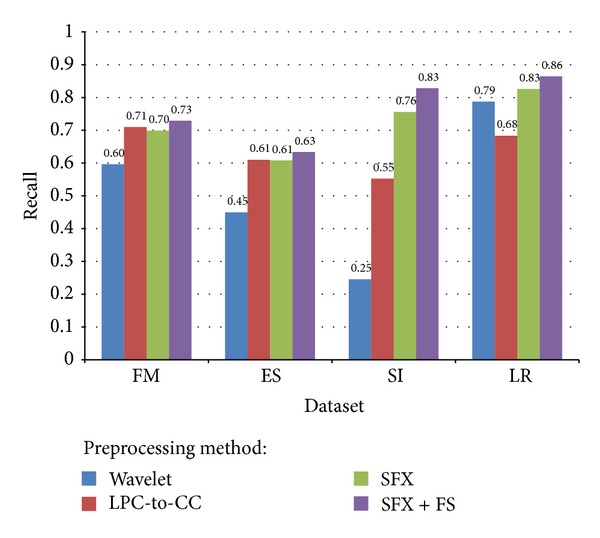
Comparison of average recall for different voice datasets and different preprocessing methods.

In a classification task, recall is a criterion of the classification ability of a prediction model to select labeled instances from training and testing datasets. A recall of score 1.0 means that each instance from that particular class is labeled to this class and all are predicted correctly, and none shall be left out [44]. Recall in this context is defined as the number of true positives divided by the total number of elements that actually belong to the positive class (i.e., the sum of true positives and false negatives, which are items which were not labeled as belonging to the positive class but should have been). The recall scores defined loosely as “of all the instances that are truly of a particular class, how many did we classify them into that class?” For example, as shown in Figure 17, 86% of instances are classified into the classes and they actually belonged to those classes. Inversely 14% is missed out. Again, the recall scores for Wavelet method are comparatively low except in the LR dataset it exceeds that of LPC-to-CC method. Having a low recall score means the classifier is conservative. SFX-FS is outperforming the rest of the methods in terms of recall scores. The comparison is shown in Figure 17.

#### 5.3.4. *F*-Measure

*F*-measure is the harmonic mean of precision and recall, that is,

(37) F-measure=21/Precision+1/Recall=2·Precision·RecallPrecision+Recall.

It is also known as balanced *F* score or *F*-measure in tradition, because recall and precision are equally weighted. The general formula for *F*_*β*_ measure is

(38) Fβ=1+β21/Precision+β2/Recall=(1+β2)·Precision·Recallβ2·Precision+Recall.

**Figure 18:**
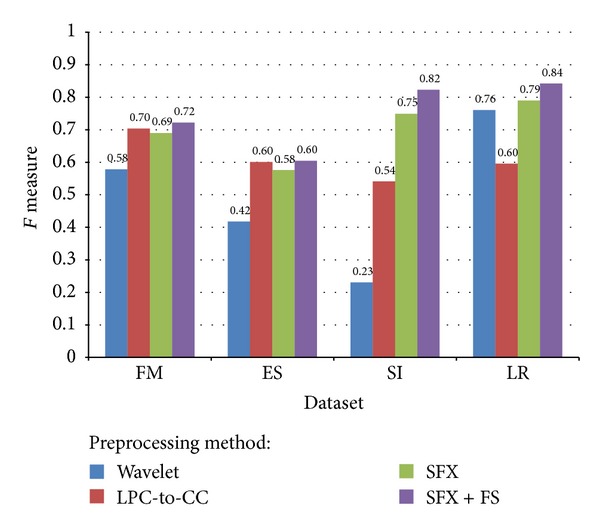
Comparison of average *F*-measure for different voice datasets and different preprocessing methods.

As mentioned before, precision and recall scores should be taken into account simultaneously because they have a strong relation essentially. Consequentially, both are combined into a single measure, which is *F*-measure. Other complicated combinations of precision and recall include but are not limited to the weighted harmonic mean of precision and recall (*F*_*β*_), and the geometric mean of regression coefficients, and Informedness and Markedness (Matthews correlation coefficient [45]). In our experiments, we only concern *F*_1_-measure. *F*_1_ measure is a derived effectiveness measurement. The resultant value is interpreted as a weighted average of the precision and recall. The best value is 1 and the worst is 0.  Figure 18 shows a comparison of average *F*_1_ measure for different voice datasets and different preprocessing methods. SFX-FS shows superior *F*_1_ score in datasets SI and LR; it duels with LPC-to-CC in simple datasets like FM and ES.

#### 5.3.5. ROC

A Receiver Operating Characteristic (ROC) is generated by plotting True Positive Rate (TPR) verse False Positive Rate (FPR) with many value settings of threshold. It is a graphical plot which illustrates the performance of sensitivity and specificity. TPR is also known as sensitivity, and FPR is one minus the specificity or true negative rate. A ROC space is defined by FPR and TPR as *x* and *y* axes, respectively, with the coordinate (0,1) representing the best prediction result. The area-under-curve (AUC) statistic of ROC is commonly used in machine learning and data mining community for model comparison. The AUC is an equivalent and simple replacement of ROC curve.

**Figure 19:**
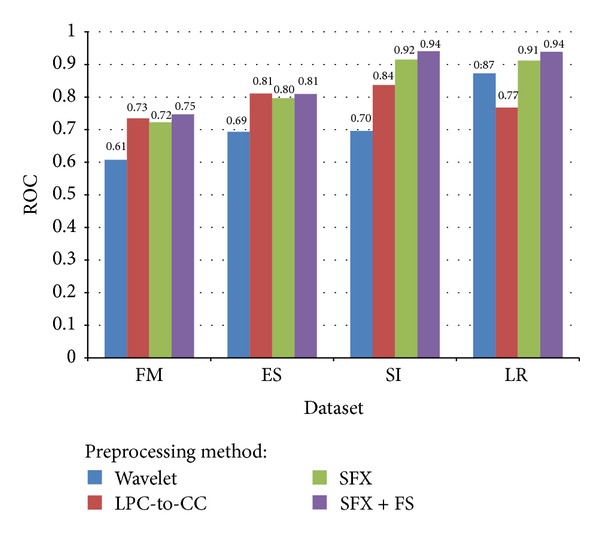
Comparison of average ROC AUC for different voice datasets and different preprocessing methods.

ROC is useful for gaining insight into the decision-making ability of the model—how likely is the classification model to accurately predict the respective classes? The AUC measures the discriminating ability of a classification model. The larger the AUC, the higher the likelihood that an actual positive case will be assigned a higher probability of being positive than an actual negative case. The AUC measure is especially useful for datasets with unbalanced target distribution (one target class dominates the other). A comparison in terms of ROC AUC which is normalized to [0, 1] for different voice datasets and different preprocessing methods is shown in Figure 19. Again, they show similar performance results to those in *F*_1_ measures. SFX + FS perform equally well in SI dataset and LR dataset with 0.94 AUC; it is slightly higher than SFX and LPC-to-CC in FM and ES datasets. Wavelet has the lowest AUC in all datasets except LR where it is better than that of LPC-to-CC.

#### 5.3.6. Aggregated Results

The final results that are averaged and aggregated, from the individual results tested by using different datasets and different classification algorithms, are shown as follows. We compare in particular various preprocessing methods against a collection of performance indicators, as in Table 8. 

From Table 8, we can reach a conclusion that SFX with FS is indeed the most suitable preprocessing method for all types of voice datasets. It has a higher value across all performance indicators than the rest of the preprocessing methods.

The accuracy and CPU time are evaluated across different feature selection algorithms; the averaged results together with the amount of attributes before and after FS are shown in Table 9.

In Table 9, the first three FS algorithms have been widely used, and the last one is recently proposed by Fong [20]. WSA gives the second fewest number of attributes after feature selection, highest classification accuracy, and a compromising time cost with 31 seconds minimum. So to some extent WSA is a good choice of feature selection if time requirement is not a concern in training up a voice classification model. WSA is done at the cost of incurring extra time in doing the heuristic optimization on the feature subset.

Table 9 shows the overall averaged time cost of each process step applied on different datasets. Piecewise transformation and DTW need much longer time than the other processes due to the computational complexity. The time consumption by piecewise transformation is relatively long especially for complex datasets like SI and LR. Statistic measures are computed for each segment (20x) for each time-series. WSA works as a stochastic iteration model, which progressively refines the performance and is superior to the other three FS methods but comes at a certain time cost. In contrast the classification model construction times in general are very short, with an average of less than two seconds. Please see Table 10. The total time required for preprocessing voice data for classification ranges from slightly less than an hour to four hours and eighteen minutes, depending on the choice of preprocessing algorithms and complexity of the datasets. Be reminded that the reference of time consumption shown here is for training a classifier based on the given training set; once a classifier is trained, the testing is very fast that takes almost no time. Therefore, a system designer can choose the best performing algorithms in terms of accuracy and other performance quality indicators if the voice classification application is not prone to frequent update of training dataset (that means no need to build the classification model over again), and of course vice versa this implies.

## 6. Conclusion and Future Works

Human voice is referred to as one of the bodily vital signs that could be measured, recorded, and analyzed as fluctuations of amplitude of sound loudness. Voice classification constitutes to a number of biometrics techniques of which the theories have been formulated, studied, and implemented in practical applications. Traditional classification algorithms from data mining domain, however, require the input of training data to be formatted in a data matrix where the columns represent features/attributes that characterize the voice data, and the rows are the instances of the voice data. Each record must have a verdict known as predicted class for training data. In the literature, mainly the characteristics of voice data are acquired from the frequency domain, for example, LPC, cepstral coefficients, and MFCC. Those popular preprocessing methods have demonstrated significant advantages in transforming voice data which is in the form of time-series to signatures in the frequency domain. There exist possibilities that some useful attributes can be harvested from the time domain considering the temporal patterns of voice data that are supposedly distinctive from one another. A challenge to overcome is its expensive computational cost of time and large search space in the time domain.

Considering the stochastic and nonstationary nature of human voice, a hybrid data preprocessing methodology is adopted in voice classification in this paper, where combined analysis from both frequency and time domain is included. In particular, a time domain feature extraction technique called Statistics Feature Extraction (SFX) is presented. SFX utilizes piecewise transformation that partitions a whole time-series into segments and statistics features are extracted subsequently from each piece. Simulation experiments were conducted on classifying four types of voice data, namely, Female and Male, Emotional Speech, Speaker Identification, and Language Recognition into different groups by using SFX and its counterparts (SFX and Feature Selection). The results showed that SFX is able to achieve a higher accuracy in the classification models for the four types of voice data. 

The contribution is significant as the new preprocessing methodology can be adopted by fellow researchers that will enable them to build more accurate voice classification model. Besides, the feature selection result proves that a metaheuristic feature selection algorithm called Wolf Search (WSA) can achieve a global optimal feature subset for highest possible classification accuracy. As there is no free lunch in the world, WSA costs considerable amount of computational time. 

The precision of piecewise transformation segmentation can be one of the future works. If the number of segments is too large (low resolution in time-series modeling), then it will lead to the low accuracy of feature extraction; if the window is too small (with very refined resolution), then a lot more computational costs are incurred. Although calibration was done beforehand for calculating the ideal segment length for subsequent processing, this again contributes to extra processing time, and the calibrated result may need to be refreshed should the natures of the voice data evolve. Some dynamic and incremental methods are opted for solving this calibration problem for estimating the correct length of segments. Furthermore the segment lengths can be variables that cope with the level of fluctuation of the voice data, dynamically.

## Figures and Tables

**Table 1:** The piecewise segment statistics feature extraction.

Attribute	1	2	3	*⋯i* *⋯*	20
Gradient	Grad 1	Grad 2	Grad 3	*⋯⋯*	Grad 20
RSS	RSS 1	RSS 2	RSS 3	*⋯⋯*	RSS 20
Resstd	Resstd 1	Rresstd 2	Resstd 3	*⋯⋯*	Resstd 20
Volmean	Volmean 1	Volmean 2	Volmean 3	*⋯⋯*	Volmean 20
Volstd	Volstd 1	Volstd 2	Volstd 3	*⋯⋯*	Volstd 20

**Table 2:** Distributions of classes in different datasets.

Dataset name	No. of classes or labels	Notes
FM	2	Female and male
ES	4	Happiness, anger, sadness, and neutral
SI	16	16 different speakers
LR	3	Cantonese, English, and Mandarin

**Table 3:** The numbers of attributes associated with datasets and instances for training and testing by various preprocessing methods.

Preprocessing method	FM	ES	SI	LR
Wavelet	50	50	50	50
LPC-to-CC	10	10	10	10
SFX	74	68	88	75
SFX + FS	20	53	20	32
No. of instances for training	258	179	564	600
No. of instances for testing	172	160	272	150

**Table 4:** List of standard classification algorithms used in our experiment.

Standard classification algorithm type	Algorithm
Bayes	NaiveBayes

Functions	LibSVM
	Multilayer perceptron
	SMO

Meta	Bagging

Rules	Conjunctive rule
	Decision table
	FURIA
	JRip/RIPPER
	NNge
	OneR
	PART

Decision Trees	BF tree
	FT
	J48/C4.5
	LMT
	NB tree
	Random forest
	Random tree
	REP tree

**Table 5:** Optimal FS methods for each dataset.

FS accuracy %	FM				ES				SI				LR			
Feature selection method	CFS	ChiS	MRMR	WSA	CFS	ChiS	MRMR	WSA	CFS	ChiS	MRMR	WSA	CFS	ChiS	MRMR	WSA
Original no. of attributes	74	74	74	74	68	68	68	68	88	88	88	88	75	75	75	75
No. of attributes after FS	8	55	30	20	17	53	30	21	20	71	30	29	32	32	30	31
Classification algorithm																
J48	63.1783	64.7287	63.5659	67.4419	61.25	59.375	57.5	64.375	73.1618	66.1765	74.6324	64.7059	94	92.3333	70	94
BFTree	71.7054	68.9922	62.0155	76.3566	58.125	56.25	65	65.625	83.0882	79.7794	85.6618	81.25	93.3333	91.6666	68	93.3333
FT	74.031	79.0698	72.8682	82.5581	64.375	78.125	65.625	88.75	97.4265	88.9706	93.75	88.2353	72.6667	71	69.3333	76
LMT	63.5659	92.4419	73.6434	97.6744	60.625	83.125	70	88.125	96.3235	92.4465	91.9118	87.8676	86.6667	85	66.6667	86.6667
NBTree	71.7054	63.1783	63.1783	70.155	51.25	59.375	67.5	66.25	n/a	n/a	n/a	n/a	67.3333	65.6666	66.6667	68
RandomForest	73.6434	70.5426	67.8295	72.8682	61.875	74.375	71.25	73.125	90.4412	74.2647	81.9853	81.9853	90.6667	89	70.6667	90.6667
RandomTree	64.7287	59.6899	55.814	70.155	45	66.25	57.5	67.5	69.1176	61.7647	69.4853	63.9706	85.3333	83.6666	84.6667	85.3333
REPTree	72.093	72.093	67.4419	73.6434	56.875	61.25	60	64.375	84.9265	84.1912	79.4118	83.4559	94.6667	93	66.6667	94.6667
ConjunctiveRule	55.814	55.814	48.8372	65.8915	54.375	55	55	56.25	n/a	n/a	n/a	n/a	66.6667	65	65.3333	66.6667
DecisionTable	70.155	70.155	53.876	68.6047	57.5	56.25	61.875	52.5	67.2794	58.8235	63.2353	59.5588	93.3333	91.6666	77.3333	93.3333
FURIA	71.7054	77.1318	63.9535	74.8062	62.5	68.75	64.375	53.125	80.5147	62.5	78.6765	86.3971	84	82.3333	70	84
JRip	72.8682	71.7054	67.8295	73.6434	66.25	54.375	58.125	55	74.2647	34.9265	72.0588	65.4412	98	96.3333	66.6667	98
NNge	68.9922	66.6667	55.814	63.5659	53.125	46.25	52.5	50.625	92.6471	91.1765	92.2794	81.25	87.3333	85.6666	69.3333	87.3333
OneR	50.3876	50.3876	54.2636	54.2636	54.375	54.375	54.375	61.25	55.1471	55.1471	49.2647	49.2647	93.3333	91.6666	54	93.3333
PART	64.3411	68.6047	62.4031	68.6047	58.75	56.875	58.125	70.625	73.5294	67.2794	80.5147	71.3235	94	92.3333	69.3333	94
NaiveBayes	70.9302	64.7287	67.4419	67.0543	64.375	68.75	58.75	58.75	95.2206	76.8382	86.0294	72.0588	78.6667	77	64	78.6667
Bagging	73.2558	75.1938	70.155	75.969	63.75	64.375	68.75	53.125	89.3382	85.6618	84.9265	86.3971	93.3333	91.6666	66.6667	94.6667
LibSVM	66.6667	87.5969	63.5659	89.9225	n/a	n/a	n/a	n/a	n/a	n/a	n/a	n/a	57.3333	55.6666	46.6667	57.3333
MultilayerPerceptron	68.6047	79.0698	72.4806	89.5349	63.75	70.625	61.875	66.875	91.9118	93.75	93.75	86.3971	100	98.3333	70	100
SMO	72.4806	77.907	73.6434	79.4574	76.875	75.625	61.875	67.5	93.0147	93.75	93.3824	80.5147	98	96.3333	76	99.3333
Mean accuracy %	68.04263	70.78489	64.03102	**74.108535**	59.73684	63.65132	61.57895	**64.40789**	82.78547	74.55568	80.64448	*75.88668 *	86.43333	84.76663	67.90001	**86.76667**
Time (s)	0.78	2.867	3.56	*31.275 *	1.03	3.328	1.439	***441.476***	1.91	3.815	3.26	***3585***	1.39	2.17	4.8	***906***

**Table 6:** Strength of agreement of Kappa statistic.

Kappa	Agreement	Interpretation
<0	Less than chance agreement	Poor
0.01–0.20	Slight agreement	Slight
0.21–0.40	Fair agreement	Fair
0.41–0.60	Moderate agreement	Moderate
0.61–0.80	Substantial agreement	Substantial
0.81–1.00	Almost perfect agreement	Almost perfect

**Table 7:** Definitions of precision and recall terms.

	Actual Class (Observation)	
Predicted Class (Expectation)	TP (True Positive) Correct Result	FP (False Positive) Unexpected Result
	FN (False Negative) Missing Result	TN (True Negative) Correct Absence of Result

**Table 8:** Overall Averaged Performance Comparison of Pre-processing Methods.

Average performance	Pre-processing methods			
	Wavelet	LPC-2-CC	SFX	SFX + FS
Accuracy %	52.67789	63.70274	72.72099	**76.72044**
Kappa Statistics	0.335301	0.490773	0.58568	**0.643008**
Precision	0.515225	0.652195	0.730832	**0.771412**
Recall	0.519617	0.638896	0.721978	**0.763601**
F-measure	0.496758	0.610196	0.701144	**0.747919**
ROC	0.717222	0.787528	0.836521	**0.859025**

**Table 9:** Overall averaged performance comparison of ensemble feature selections.

FS	No. attributes from frequency domain	No. attributes from time domain	Total no. attributes	No. attributes after FS	Average CPU time (s)	Av. Acc. %
CFS	10	66	76	19	1.28	74.25
ChiSq	10	66	76	52	3.05	73.44
MRMR	10	66	76	30	3.26	68.54
WSA	10	66	76	25	1240 (min. 31)	75.29

**Table 10:** Overall averaged time cost comparison.

Time	Preprocessing				FS		Build Model	Total
Dataset	LPC2CC	DS	DTW	Piecewise				
FM	10 s	5 m 23 s	15 m 3 s	32 m	CFS	0.78 s	1.13 s	52 m 37.9 s
					ChiSq	2.867 s		52 m 40 s
					MRMR	3.56 s		52 m 40.7 s
					WSA	31.275 s		53 m 18.4 s

ES	9.5 s	9 m 35 s	21 m 38 s	1 h 13 m	CFS	1.03 s	1.25 s	1 h 44 m 24.8 s
					ChiSq	3.328 s		1 h 44 m 27.1 s
					MRMR	1.439 s		1 h 44 m 25.2 s
					WSA	441.476 s		1 h 51 m 45.2 s

SI	15.8 s	25 m 6 s	38 m 23 s	2 h 14 m	CFS	1.91 s	1.7 s	3 h 17 m 48.4 s
					ChiSq	3.815 s		3 h 17 m 50.3 s
					MRMR	3.26 s		3 h 17 m 49.8 s
					WSA	3585 s		4 h 17 m 31.5 s

LR	13.4 s	16 m 48 s	42 m 45 s	1 h 57 m	CFS	1.39 s	1.56 s	2 h 56 m 49.4 s
					ChiSq	2.17 s		2 h 56 m 50.1 s
					MRMR	4.8 s		2 h 56 m 52.8 s
					WSA	906 s		3 h 11 m 54 s
